# Placenta Prævia, and Other Cases

**Published:** 1869-08

**Authors:** B. H. Cheney

**Affiliations:** Joliet, Ill.


### PLACENTA PRÆVIA, AND OTHER CASES.

---

Reported by B. H. CHENEY, M.D., of Joliet, Ill.

---

Three cases of placenta prævia have recently occurred in my practice; a much larger number proportionally than is usual. The cases present nothing remarkable either in their pathology or treatment; still, the nature of this dangerous complication of labor is always of such interest and importance that they will perhaps bear a brief rehearsal.

### PLACENTA PRÆVIA LATERALIS.

CASE I. Mrs. B., aged 34 years, multipara, American, temperament leucophlegmatic; no hemorrhage in previous labors. A midwife attended her in her present confinement. I subsequently learned that there was considerable hemorrhage at the commencement of the labor, but that it soon ceased; the pains being frequent and strong, and the delivery rapid. As soon, however, as the child was born, alarming hemorrhage again set in, and a physician was sent for in great haste. The child had been born some 15 minutes when I reached the house. Found the patient bloodless; lips as pale as her cheek; respiration extremely faint and scarcely perceptible; pulse imperceptible at wrist, faintly perceptible over heart; flooding profusely. On examination, found umbilical cord had been torn off at its placental insertion by the midwife's traction upon it. About one-fourth or one-third of the placenta was lying unattached over the os; the remainder firmly adherent to the left wall of the cervix. I at once detached it, as is usual in such cases, by passing the fingers under it, being careful to secure and remove the whole organ. Then dashing ice-water, at once, upon the abdomen, the uterus promptly contracted, and hemorrhage ceased. Brandy and ammonia were given at short intervals, to the first dose of which a portion of ergot was added, as a precautionary measure. At the end of two hours, patient showed signs of returning consciousness, and from that time continued to improve. Recovered; child living.

The history of this labor is what would have been expected under the existing conditions. There was some flooding when the dilating pains detached that portion of the placenta immediately over and around the os; but the presentation was, fortunately, natural, the uterine contractions frequent and strong, and the labor quick: the child's head, by its pressure, assisted in arresting the incipient hemorrhage, which, however, of course, returned at once on the birth of the child and the cessation of uterine contraction. I neglected to ascertain if any hemorrhage had occurred in this patient during the latter part of her pregnancy. If so, it must have been so slight as to excite no alarm, as no physician was consulted.

PLACENTA PRÆVIA CENTRALIS.

CASE II. Mrs. G., aged 37 years, Irish, robust and stout, temperament bilious, multipara. Previous labors natural.

*August 18th.* Was called to stop an attack of flooding; patient not expecting her confinement for two weeks or more. Some four weeks ago, had an attack of hemorrhage, which has recurred from time to time since. Present condition—pulse regular; respiration good; loss of blood considerable, but not alarming; no sign of commencing labor; os uteri so closed that I could not diagnose whether this was a case of placenta prævia or of detached placenta. The clinical history, however, led me to presume the former. Prescribed rest in recumbent posture, cool drinks, acid. sulph., etc.

*August 21st.* Was called at 7.30 P.M. Waters had broken at 4 P.M. Os uteri dilated to size of a quarter dollar piece, extremely rigid. Found the placenta attached directly over it; the pains very irregular, undecided, and inefficient, but the hemorrhage alarming, and patient prostrated. Used ice locally, and gave ammonia and whiskey. Dr. A. W. Heise in consultation. The os being still rigid, with no contractile pains, and flooding continuing, we decided to remove the placenta, at once, if possible. Found it to be central; os so rigid that only pieces broken up could be removed at a time. Removed as much as possible, and inserted tampon. One and a-half hour later profuse flooding. Removed tampon, and more of placenta. Os still rigid: no contractile pains. Presentation—left shoulder, with elbow down, back of child anterior.

On further consultation, determined to administer chloroform to relax the os, and enable us to turn. Patient has been taking whiskey constantly; to the last two doses of which was added fl. ext. secal. cornut. Administered chloroform at 2 A.M. Dr. Heise turned, bringing down feet. Allowed patient to come out from anæsthesia, and repeated whiskey and ergot. Beginning traction, slight labor pains came on, and labor was completed with no difficulty. Removed remaining pieces of placenta and blood-clots from uterus, and applied ice-water to abdomen. Contraction took place, and all hemorrhage ceased. Child, of course, stillborn. Mother, although exhausted by fearful loss of blood, recovered, with no untoward symptom.

The prominent features of the above case—the rigidity of the os, the total absence of contractile pains, etc.—render the reasons for attempting to detach and remove the placenta, as the first procedure, so evident, that it is not necessary to state them in detail. My limited experience and my judgment would not lead me to adopt, as a general rule, the plan so strongly advocated by its able and distinguished proposer, Prof. Simpson, of Edinburgh, *viz.*, that of artificial detachment and delivery of the placenta before the child. Still, I am aware that the Professor does not recommend this as a method applicable to all cases; and there may be some in which it will be found feasible, if not advisable. Believing that the hemorrhage proceeds from the bloodvessels of the uterus (the utero-placental), and not, unless secondarily, from those of the placenta, the indications above all others seem to be—1st. To empty the cavity of the womb; and 2d. To secure its permanent contraction. The test of clinical experience, and not theorizing, is to decide by what method these results can be quickest and safest attained.

### PLACENTA PRÆVIA CENTRALIS.

CASE III. Mrs. D., aged 22 years, multipara, German, leucophlegmatic, actress. Was called at 10 A.M. Found patient at beginning of labor; pains weak and inefficient; flooding with each one. On vaginal examination, found os nearly dilated, soft, and yielding; placenta attached directly over mouth of womb; presentation, cephalic. Pains had begun only about an hour previous. The hemorrhage was increasing, and the patient rapidly losing strength. Accordingly, in the presence of Dr. Harwood, who was called in counsel, I introduced the hand; and, finding it would be difficult and occasion loss of time to get around the placenta, as is usually recommended, I passed directly through it, turned, and delivered at once by the feet, removing the placenta immediately afterwards. Cold water to the abdomen at once induced contraction of the uterus, and the hemorrhage did not recur. The whole delivery did not occupy five minutes, but the child was *in articulo mortis*, gasping but a few times. Persistent efforts and trial of every means for its restoration, for three-quarters of an hour, were wholly unavailing. Mother recovered well and rapidly.

Placenta prævia is variously estimated by different authorities to occur from 1 in 500 to 1 in 1000 cases. The average of statistics, from various sources, give as the result 1 in 3 of the mothers, and over ½ of the children lost. The resort, in these cases, to premature delivery, as a prophylactic measure, as recommended by Prof. Thomas (*vide American Journal of Obstetrics*, for May, 1868), would, doubtless, materially diminish this mortality. As Dr. Thomas says: "By resorting to this measure, we should be dealing with a woman who is not exhausted by repeated hemorrhages, the obstetrician would be in attendance at the commencement of the labor; and he would be able, by hydrostatic pressure, to control flooding, while the same pressure accomplished rapidly and certainly the first part of the labor."

The case of Mrs. G., No. II., is one in point. Premature delivery, by the use of Barnes' dilators, would very probably have prevented the occurrence of such profuse hemorrhage, and its attendant dangers.

Dr. Thomas, in the article referred to, gives as the reasons for the great mortality in placenta prævia—1st. Hemorrhage, occasioned by dilatation of cervix; 2d. Repeated hemorrhages, occurring during ninth month; 3d. Profuse flooding at commencement of labor. To these, it seems to me, should be added the unusually large proportion of unnatural presentations which occur in this complication of labor, rendering the introduction of the hand and turning necessary, aside from the malposition of the placenta and the hemorrhage.

### LATENT METRITIS. (?)

Mrs. H., aged 38 years, multipara, Irish, bilious habit. This patient lived five miles in the country; and I was not called until she had been in labor 14 hours. On arriving at the house, found delivery completed, and patient in very favorable condition. Left her with a few general directions. On the fourth day thereafter, the husband came to town, and informed me that his wife was "not doing well;" lochia were suppressed; had fever, etc. Prescribed a poultice of flax-seed and camphor to vulva, warm applications, etc., laxatives, and spts. mindereri. Fifth day, no better; was asked to visit her. Found her with no fever, pulse regular and soft, skin moist and natural. Bowels had moved; urine passed freely; no abdominal tenderness; whole condition except suppression of lochia, apparently favorable. Vaginal examination revealed nothing abnormal; parts were not heated nor dry; nor was there any indication of inflammatory action. Lochia completely suppressed since third day, on which, I now learned, she had been up and about her room a good deal, having no one to attend her. Lacteal secretion good and abundant. The patient was firmly persuaded that she was going to die. Her mental condition was much like that in hysteria, without the convulsive seizures; a state of perfect *melancholia.* She was convinced that her husband and his brother were determined to poison her, and would take neither nourishment nor medicine from them. The hygienic conditions in her surroundings—room, attendance, etc.—were as bad as they could be. On the eighth day, at evening, she jumped from the bed, and seizing her brother-in-law, shook him violently; whether aggravated thereto, or simply from frenzy. I could not learn. This was the only approach to convulsion which she manifested. Saw her at midnight. Heart's action was then quite irregular; frequent cessation of pulsation, etc., but no sign of organic disease. Excessive wakefulness had been a prominent symptom. Persuaded her to eat some beefsteak and bread, and drink some tea, after which she had a natural and refreshing sleep of two hours. She had for several days persistently refused all nourishment, except a few dry crackers, and had no sleep for two days and nights. She awoke in the same state of profound melancholia, and continued in much the same condition—refusing, finally, to take anything at all, nourishment or medicine; sitting moody and silent, or muttering to herself—until the twelth day, when she died.

Although a firm persuasion of the certainty of death is, in any disease, almost enough to ensure its fatality, or, perhaps, existing as a consequence, shows that a fatal issue is to take place, yet, this conviction alone is not enough to *cause* death. There must, therefore, have been some pathological condition behind all this. I do not pretend to decide what it was, but have ventured to suppose that it might have been a latent developed metritis (what our German brethren would term *dunkel ausgesprochen*), consequent upon suppression of the lochia, and producing a toxæmic condition. A *post mortem* might have decided this point, but could not be obtained. Cases of puerperal mania, resulting from the same cause, and manifesting symptoms similar to the above, have, doubtless, occurred to almost every one engaged in obstetric practice. Their true pathology seems to be not well understood. There is, probably, some depravation of the blood connected with it.

### ATRESIA VAGINÆ.

N. H., aged 3 months. The mother informed me that a few days after the birth of this infant she noticed a "string of clotted blood" between its labia vulvæ, and on looking more closely found no appearance of any vaginal orifice. I found, upon examination, the parts normal in every respect, except complete occlusion of the vagina; its site being covered by skin in no way different from ordinary integument, except that it was lighter, and marked perpendicularly by a faintly perceptible raphé. The clotted blood spoken of by the mother, and the existence of the raphé, led me to conclude that this occlusion was traumatic, and not due to any error of development; and, further, that the vagina probably existed normally behind this wall of integument. I accordingly divided it with a bistoury, and gently introducing a female catheter a short distance, found the canal pervious, as I had anticipated. A tent introduced to prevent reunion of the edges of the wound completed the curve.

Had there been complete imperforation of the vagina in this case, I should, of course, have advised to defer any operation for reöpening the canal, or forming an artificial vagina, until the occurrence of puberty should have demonstrated the existance of ovaries, and accumulation of menstrual fluid the presence of a uterus.

It is difficult to assign any cause which could operate *in utero* to occasion traumatic closure of the vagina. The skin was, however, too perfect to have grown in the few days following the child's birth; and, on the other hand, the presence of the clotted blood is difficult to account for, if the deformity be presumed to be congenital.

Dr. A. W. Heise, of Joliet, has related to me, from memory, two cases of interest, of which the following are the leading features:—

ABORTION AT FOURTH MONTH, FOLLOWED BY PREMATURE DELIVERY OF SECOND FŒTUS AT SEVENTH MONTH.

CASE I. Mrs. C., aged 32 years, German, mother of four children. Landed in the U. S. three months since. The husband of this woman called upon Dr. H., and requested him to prescribe for his wife who was flooding. On visiting the patient, found her apparently threatened with miscarriage; abdomen enlarged, as though pregnant. The patient, however, stoutly maintained that she was not, and could not be, pregnant; stating that, three months previously, on the passage across the Atlantic, she had miscarried with a fœtus of about four months, according to her own calculation of her pregnancy, and to the statement of the midwife who attended her upon shipboard; that she had herself seen the child after its birth, and there could be no mistake about it; that she had been flowing, more or less, at intervals, ever since, and had had no sexual intercourse since that time. The Doctor accordingly concluded that this was a case of retention of some of the secundines, or of polypus, or mole. The woman was having expulsive pains, and, upon vaginal examination, the Doctor discovered a mass advancing, though he did not recognize the part presenting sufficiently to diagnose what it might be. The patient was soon delivered, however, of a fœtus completely doubled upon itself in emprosthotonos, the convexity of the spine presenting. The fœtus was apparently of the sixth month, and partially decomposed. Granting the correctness of the woman's statement, in reference to the previous abortion, the present fœtus must have lived two months after the birth of its twin. No examination was instituted as to the existence of a double uterus.

TRAUMATIC ATRESIA AND IMPERFORATION OF VAGINA.

CASE II. Mrs. A., aged 20, primipara. Dr. Heise was asked to visit this case in consultation with Dr. Bacon, of Lockport. Patient had been 24 hours in strong labor, with no progress. Dr. B. stated that he had not been able to ascertain the presentation, as he could not introduce the finger into the vagina. Dr. H., attempting an examination, found the canal completely occluded by a tense, firm membrane, about one-third of an inch from its external orifice. There was a small opening, sufficient to admit a good-sized probe, at the upper or pubic part of this membrane, but no justifiable force could make way for the finger. Through this opening the waters had discharged some 10 or 12 hours previously, since which time the patient had been in strong labor, with constant expulsive pains. The husband being asked if he had ever had complete connection with his wife, replied that "he had," but on being questioned closer, stated that he "had some time ago, but not recently." On being pressed for his knowledge of any cause for her present condition, he acknowledged that he had been married only about two months, that he had had sexual intercourse with his wife some time previous to their marriage, that finding herself *encinte*, they had procured the services of an abortionist, who had operated upon her for 15 minutes with a wire, and pronounced the operation complete; that after this operation she was sick and confined to her bed for six weeks, purulent and bloody discharges taking place from the vagina.

Inasmuch as the passage of a probe through the orifice in the membrane showed a cavity beyond it, Dr. H. hoped it might be the only obstruction. On dividing it, however, he found still others, in the form of fibrous bands, stretching obliquely across the vaginal canal, interlacing with each other, the whole length of the vagina, and very firm and strong. Carefully dividing these, the fœtal head was plainly felt in the first position, and the patient was soon delivered of a healthy child. Severe inflammation of the vagina, womb, and peritoneum supervened, and she died on the ninth or tenth day thereafter.

